# Editorial: Transgenesis and secondary metabolites: recent developments and technological challenges

**DOI:** 10.3389/fpls.2023.1276922

**Published:** 2023-08-30

**Authors:** Sumita Jha, Hengfu Yin, Chenghao Li, Guang-Long Wang

**Affiliations:** ^1^ Department of Botany, Calcutta University, Kolkata, India; ^2^ Research Institute of Subtropical Forestry, Chinese Academy of Forestry, Hangzhou, China; ^3^ School of Forestry, Northeast Forestry University, Harbin, China; ^4^ School of Life Science and Food Engineering, Huaiyin Institute of Technology, Huai'an, China

**Keywords:** plant secondary metabolites(PSMs), metabolic engineering, genome editing, E3 ubiquitin ligase, indole alkaloid biosynthesis, paclitaxel biosynthesis, natural products(NPs), transgenic cultures

Plant secondary metabolites are a diverse group of physiologically active phytochemicals derived from a complicated process of secondary metabolism in higher plants. In addition to their essential roles in plants, including acclimation, adaptation, and survival responses to various challenges presented by biotic and abiotic stressors, these metabolites also have substantial economic relevance in the pharmaceutical and cosmetic industries. In recent decades, the increasing demand for plant-derived bioactive molecules, coupled with several limiting factors in classical plant secondary metabolite production from wild plants and advancements in biotechnology and plant transgenic research, has opened up new avenues for transgenic culture-mediated secondary metabolite production. The amount of these metabolites is limited and can be significantly influenced by various factors such as plant species, tissue, developmental condition, and environmental conditions. The transgenic approach to secondary metabolite production offers a unique opportunity to create effective and sustainable production of high-value phytochemicals. While several gene-transfer technologies, *in vitro* culture systems, and biotechnological approaches are now available for transgenic culture-mediated secondary metabolite production, the success greatly relies on sensible selections of technology or approach by researchers based on their understanding of both the expected and unforeseen consequences of the approach chosen. Several medicinal plants have already been genetically transformed using effective bacterial vector systems for producing high-value phytochemicals. Economically viable large-scale production of the desired product using transgenic cultures is possible in bioreactors (Kowalczyk et al., 2022), which has opened up new opportunities for their commercial use in medicine. Furthermore, the application of cutting-edge genetic engineering techniques expands the potential of this field in a cumulative way (Sirirungruang et al., 2022; Kwan et al., 2023).

The Research Topic showcases innovative work and highlights the research progress and direction of the field, focusing on various advancements in genetic transformation, expression regulation, metabolic engineering, synthetic biology, genome editing approaches in plants, and more. This discipline is vast and continuously undergoing rapid expansion, with new avenues constantly emerging.

This Research Topic consists of five articles contributed by forty authors and we present a summary of their salient features.

## 1 Genetic engineering of paclitaxel biosynthesis

Paclitaxel is one of the most important antitumor agent, identified from *Taxus* spp, and its biotechnological production has gained attention over the years. Metabolic engineering of paclitaxel biosynthesis in *Taxus* spp., however, remains a challenging task, mainly due to the difficulty of genetic transformation and low efficiency of paclitaxel biosynthesis. In this regard, the paper by Perez-Matas et al. presents the current progress in enhancing paclitaxel production using genetic engineering techniques. The review primarily focuses on the overexpression of taxol biosynthetic genes or transcription factors in cell cultures and roots of different *Taxus* species, as well as synthetic production in a heterologous system. The authors highlight the difficulties encountered in the genetic transformation of *Taxus* species and emphasize that the incomplete understanding of the biosynthetic pathway of paclitaxel hinders further progress and applications in this area. Despite these challenges, recent advancements in whole genome sequencing of *Taxus* spp. and enzymatic engineering have shown promise, suggesting that heterologous systems may be favored for future biotechnological production of paclitaxel.

## 2 Indole alkaloid biosynthesis for secondary metabolism

The plant *Baphicacanthus cusia* (Acanthaceae) is pharmacologically important for its accumulation of indole alkaloids, including indigo and indirubin. Indole alkaloid biosynthesis, which plays a vital role in various plant functions, has been extensively studied, with indole serving as a key precursor for secondary metabolites. In their research, Guo et al. proposed that the tryptophan synthase alpha-subunit (TSA) is a critical enzyme responsible for catalyzing indole synthesis in the biosynthesis of indole alkaloids. The researchers identified, characterized, and verified the biological functions of BcTSA (a homologous gene from *Baphicacanthus cusia*), which was identified based on the transcriptome of *B. cusia*. Furthermore, the team demonstrated that the recombinant BcTSA enzyme was capable of catalyzing the conversion of IGP (indole-3-glycerol phosphate) to indole. Through overexpression of *BcTSA*, they were able to enhance the accumulation of indole alkaloids in hairy roots of *Isatis indigotica*, further highlighting the importance of BcTSA in indole alkaloid production.

## 3 Perspectives of genome editing in secondary metabolites biosynthesis

Mipeshwaree Devi et al. present a comprehensive discussion on the current trends in plant metabolic engineering. They offer an overview of recent technological updates for engineering secondary metabolic pathways, with a particular focus on the CRISPR-Cas system. The authors highlight the wide utilization of tissue-specific genome editing in metabolic engineering since secondary metabolites tend to accumulate in various parts of plants. They provide detailed insights into recent progress made in genome editing for the production of secondary metabolites using CRISPR/Cas9 in 28 different plant species,covering grain and oil crops (e.g., soybeans, rice, peanuts), vegetables (e.g., tomatoes, potatoes), ornamental flowers, and others. The paper covers crucial information, such as the target gene, the promoter used, the type of editing, the method of delivery for the CRISPR/Cas system, and the respective secondary metabolites that have been successfully reported in these species.

## 4 Hairy root culture technology for secondary metabolites production

The use of hairy root cultures in secondary metabolite production has been reported in numerous species from diverse families, and various biotechnological interventions are now feasible. In their comprehensive review, Biswas et al. focus on the Solanaceae family, one of the largest and most important families of flowering plants, and discuss associated strategies for enhancing secondary metabolite accumulation, including the use of bioreactors. The review covers the production and elicitation of a large number of pharmacologically important secondary metabolites in Solanaceous hairy root cultures. Additionally, the authors explore the utilization of Solanaceous hairy roots for producing antigens used in vaccine development, functional monoclonal antibodies, and expression of recombinant proteins.

## 5 E3 ubiquitin ligases in strawberry

Strawberry (*Fragaria × ananassa* Duch.) is an economically and nutritionally important fruit crop. Strawberry is also recognized as a model plant for studying fruit development and ripening. In their research, Jiang et al. conducted a genome-wide characterization analysis of *U-box E3 ubiquitin ligase* genes in cultivated strawberries. The study focused on various aspects, including gene location, gene structure, evolutionary analysis, functional analysis, and protein-protein interaction analysis. The findings of the study provide comprehensive information about the *Fau-box E3 ubiquitin ligase* genes and shed light on their functions in fruit ripening and response to abiotic stresses in strawberries.

## Author contributions

SJ: Conceptualization, Writing – original draft, Writing – review & editing. HY: Conceptualization, Writing – review & editing. CL: Conceptualization, Writing – review & editing. G-LW: Conceptualization, Writing – review & editing.
